# Kidney Failure among Patients with Takotsubo Syndrome or Myocardial Infarction: A Retrospective Analysis

**DOI:** 10.3390/jcdd9060186

**Published:** 2022-06-09

**Authors:** Verena Bill, Ibrahim El-Battrawy, Marvin Kummer, Andreas Mügge, Assem Aweimer, Michael Behnes, Ibrahim Akin

**Affiliations:** 1 Department of Cardiology, Wiesbaden Hospital, 65189 Wiesbaden, Germany; verena.bill@umm.de; 2 Bergmannsheil Bochum, Medical Clinic II, Department of Cardiology and Angiology, Ruhr University, 44789 Bochum, Germany; andreas.muegge@bergmannsheil.de (A.M.); assem.aweimer@bergmannsheil.de (A.A.); 3 First Department of Medicine Cardiology, University Medical Centre Mannheim (UMM), Faculty of Medicine Mannheim, University of Heidelberg, DZHK (German Center for Cardiovascular Research) Partner Site Mannheim, 68167 Mannheim, Germany; marvin.kummer@studi.uni-heidelberg.de (M.K.); michael.behnes@umm.de (M.B.); ibrahim.akin@umm.de (I.A.)

**Keywords:** cardiomyopathy, takotsubo, heart failure, death, kidney, stress, mortality, predictors, arrhythmias, coronary syndrome

## Abstract

**Background:** Takotsubo syndrome (TTS) is a syndrome with ambiguous pathophysiology. Impaired kidney function (KF) seems to impact the outcome of patients with TTS. We hypothesized that KF worsens the outcome among TTS patients and furthermore, TTS patients with concomitant KF experience more adverse events compared to myocardial infarction (MI) patients with concomitant KF. **Methods and Results:** This retrospective single-center study comprised two groups (cohorts) of patients including patients with TTS and concomitant KF (*n* = 61, 27.1%) and patients with MI and concomitant KF (*n* = 164, 72.9%). The clinical outcomes were delineated as short-term outcomes defined as in-hospital adverse events during index hospitalization and long-term outcomes defined as adverse events over five-year clinical follow-ups. All-cause mortality, stroke, cardiopulmonary resuscitation (CPR), life-threatening arrhythmias, need for respiratory support, and cardiogenic shock with subsequent use of inotropic agents during index hospitalization were denoted as in-hospital adverse events. All-cause mortality, rehospitalization due to heart failure, stroke, thromboembolic events, and the recurrence of primary pathology (TTS and MI) were analyzed during five-year follow-ups after index hospitalization. A higher mortality rate was noted among TTS patients with KF compared to TTS without KF. In addition, in-hospital event rates in patients with TTS and concomitant KF compared to MI and concomitant KF were comparable with the exception of a higher rate of respiratory support in TTS patients. The mortality rate was significantly higher among patients with TTS and KF at 4 years (29.5% vs. 15.9%, *p* = 0.02) and 5 years (34.4% vs. 20.7%, *p* = 0.03) in comparison to patients with MI and concomitant KF. In contrast, the rate of re-hospitalization related to heart failure was higher at 30 days, and at one-, four-, and five-year follow-ups in patients suffering from MI and KF compared to TTS and concomitant KF. Additionally, the recurrence of MI after 4 and 5 years was higher than the recurrence of TTS (4.9% vs. 15.2%; 4.9% vs. 16.5%). There were no differences in life-threatening arrhythmias and stroke in both groups. **Conclusions:** Patients with TTS and concomitant KF have higher all-cause mortality when compared to MI and concomitant KF. The mechanisms responsible remain to be determined.

## 1. Introduction

Takotsubo syndrome (TTS) is transient ventricular ballooning with no signs of significant coronary artery disease [1]. In TTS the left ventricle (LV) is frequently affected predominated by apical ballooning; however, other parts of the heart could be affected such as the mid-ventricular [2], basal, or focal parts. The involvement of the right ventricle has additionally been reported as having an impact on the outcome [3,4,5].

A bevy of stressors (physical and/or emotional stressors) could influence the presence of TTS predominantly in postmenopausal women [6]. TTS patients may present with symptoms and diagnostic changes similar to acute coronary syndrome (ACS) [7]. However, the pathomechanisms underlying the etiology of TTS remain multifactorial, e.g., microvascular dysfunction, vasospasm, an abnormal response to catecholamines, endothelial dysfunctions, thyroid dysfunctions, and genetic factors [8,9,10,11,12]. In TTS there is a risk of atrial and ventricular arrhythmias, cardiogenic shock, stroke, or thromboembolic events, which may explain the worsened prognosis of TTS compared to a healthy population [3,4,13,14,15,16,17,18,19,20,21,22]. Furthermore, when TTS is accompanied by impaired kidney function (KF), the clinical outcomes are noted to be worse in comparison to patients with TTS alone [23,24]. In the single-center study by Ando et al., 30 patients (49%) presented a declined kidney function. Also, among patients who suffered in-hospital complications, 52% showed a lower glomerular filtration rate compared to patients without complications. A declined glomerular filtration rate was an independent predictor of complications. It is already known that KF impairs the outcome among patients with MI with an increased risk of morbidity and mortality [25]. In that study, 3210 patients with MI were retrospectively analyzed according to acute kidney injury (AKI) criteria during hospitalization. Among the cohort, 13% suffered from AKI. Not only did the in-hospital mortality increase but so did the long-term mortality. Furthermore, major adverse cardiac events increased in the presence of AKI compared to without. A head-to-head comparison of cardiac adverse events in TTS compared to MI has not yet been reported. Therefore, we wondered how KF influences the outcome in patients suffering from TTS compared to those with MI.

## 2. Materials and Methods

We report on 61 consecutive TTS patients admitted to our institution between 2003 and 2017 and 164 patients with MI between 2006 and 2010 with concomitant KF. We used Mayo Clinic criteria to include the TTS patients [26]. These criteria include the transient wall-motion abnormality in the left ventricle, regional wall-motion abnormalities that extend beyond a single epicardial vascular distribution, no obstructive coronary disease, newly detected ECG pathologies or modest elevations in cardiac troponin levels, and the absence of pheochromocytoma and myocarditis. In suspected cases of myocarditis and pheochromocytoma, magnetic resonance imaging was used to confirm the diagnosis. The MI group consisted of patients with an acute myocardial syndrome defined by TNI elevation, ECG changes consistent with either ST-elevation myocardial infarction (STEMI) or non-ST-elevation myocardial infarction, and significant coronary artery stenosis noted on coronary angiography. KF was assumed if the glomerular filtration rate (eGFR) was <60 mL/min/1.73 m^2^. The MDRD formula was used to calculate the GFR in the patient’s serum.

Two experienced independent cardiologists assisted with the angiograms, echocardiograms, and ECGs to confirm the diagnosis of TTS. The Declaration of Helsinki was taken into consideration for the study protocol and the ethical aspects were approved by the Ethics Committee of the University Medical Centre, Mannheim.

In this retrospective study, in-hospital events are defined as all-cause mortality, stroke, resuscitation, life-threatening arrhythmias, use of respiratory support, and cardiogenic shock with the subsequent use of inotropic agents during index hospitalization. Events over a follow-up of 5 years are defined as long-term all-cause mortality, rehospitalization due to heart failure, stroke, thromboembolic events, and recurrence of primary pathology (TTS or MI). Short-term and long-term events were analyzed in predefined study cohorts of TTS patients with concomitant KF and MI patients with concomitant KF. Data were obtained by chart review and/or telephone interviews with the healthcare providers involved in the case and/or relatives. If a review of medical records and the interviewing process were unable to determine the circumstances of death, it was defined as death due to an unknown cause.

## 3. Statistics

Continuous variables with normal distribution are shown as mean ± SD and those with non-normal distribution as median (interquartile range). The Kolmogorov–Smirnov test was used to assess normal distribution. Student’s *t*-test and the Mann–Whitney U-test were used to compare continuous variables with normal and non-normal distributions. Qualitative variables were analyzed by the chi-square test or Fisher’s exact test. A two-tailed Fisher’s exact test was applied in tests with a sample size of *n* = 5 or below. Fisher’s exact ratio test was used for the calculation of the odds ratios (OR) for the occurrence of events as defined by the composite endpoint.

The results are shown with 95% confidence intervals. The independent predictors with *p* < 0.10 in the univariate analysis were entered into the Cox multivariate regression to define the independent risk factors for the end-point. The results are described as hazard ratios (HRs) with 95% confidence intervals (CI). Analysis of event-free and overall survival time after TTS was performed using the Kaplan–Meier procedure, and the group differences were evaluated by a log-rank test. Statistical analysis was performed with SPSS 23; a *p* < 0.05 (two-tailed) was considered significant.

## 4. Results

### 4.1. Baseline Demographics

We analyzed the clinical and echocardiographic data of patients diagnosed with TTS and KF (*n* = 61) and patients diagnosed with MI and KF (*n* = 164).

The mean age of presentation was similar in both groups with a mean age of 70 years (69 ± 11 vs. 71 ± 11, Table 1) at the time of presentation with a female predominance in the TTS group. In contrast, there was a lower rate of cardiovascular risk factors, such as diabetes mellitus (21.3% vs. 39.6%), overweightness (26.0% vs. 46.3%), and hypertension (59.0% vs. 78.7%), in the TTS patients. Chronic obstructive pulmonary (11 patients vs. 7 patients) disease was more common in patients with TTS.

### 4.2. Clinical Presentation

There was a significant difference in diagnostic criteria such as clinical symptoms [chest pain (51.7% vs. 72.0%, *p* < 0.01), ST-elevation (26.2% vs. 45.1%, *p* = 0.01)], between the TTS vs. MI groups. T-wave inversion was more common in patients suffering from TTS (93.3% vs. 72.6%). The PQ duration was longer in patients with MI compared to the TTS patients.

Drugs on admission, such as ß-Blockers and ASS, were prescribed more often in patients with KF and ACS.

### 4.3. Echocardiographic Characteristics

**Figure 1:**
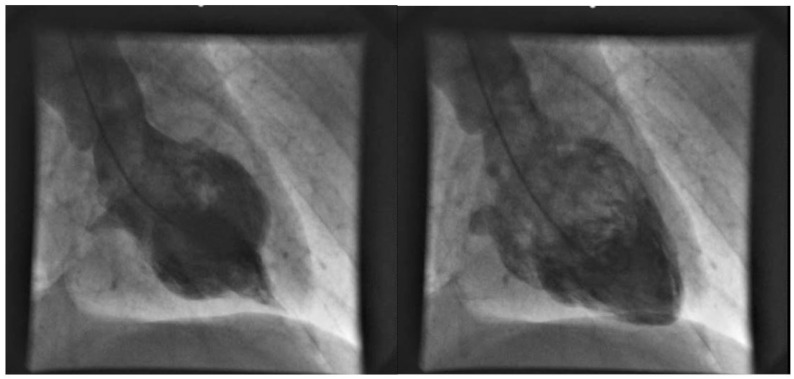
A representative sample of a female patient presenting a midventricular TTS form with no wall-motion abnormalities of the apex.

Ejection fraction (EF) during hospitalization was lower in the TTS group (37 vs. 48%, *p* < 0.01). Additionally, tricuspid valve regurgitation was more common in the TTS group (37 vs. 28%, *p* < 0.01). Mitral regurgitation seemed to be more common in the TTS patients but did not reach statistical significance. A representative example of the midventricular TTS form is shown in Figure 1.

### 4.4. In-Hospital Outcomes

There were no differences in life-threatening arrhythmias, cardiogenic shock, cardiopulmonary resuscitation (CPR), and in-hospital death in both groups (Table 2). However, the need for non-invasive and invasive ventilation and the length of stay in the intensive care unit was longer in patients with TTS (6 d vs. 3 d, *p* = 0.02; Table 2). In contrast, device implantation (ICD or pacemaker) was more common in patients suffering from MI and KF (6.6% vs. 26.8%, *p* < 0.01).

### 4.5. Long-Term Outcomes

**Figure 2:**
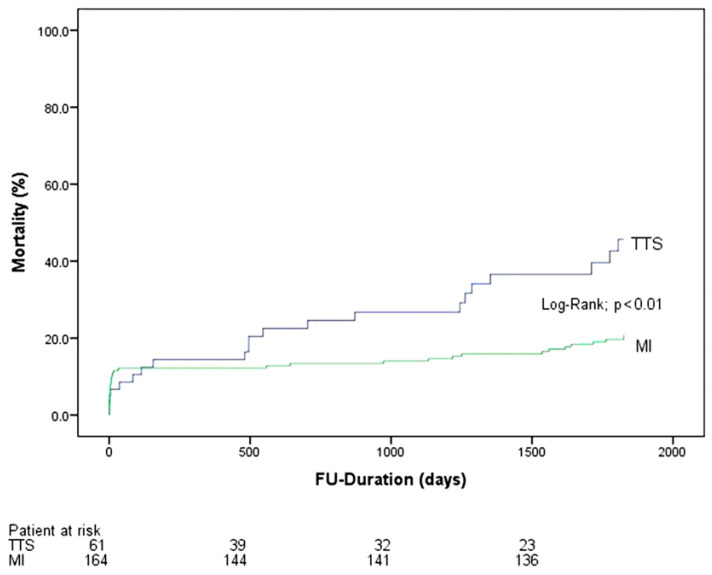
Mortality rate in patients suffering from takotsubo cardiomyopathy (TTS) and KF compared to patients with MI and kidney failure at five-year follow-up.

**Figure 3:**
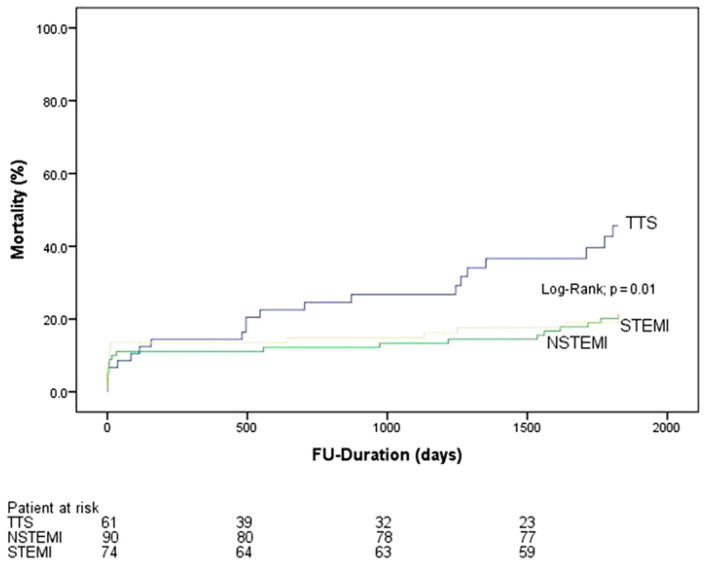
Mortality rate in patients suffering from takotsubo cardiomyopathy (TTS) and KF compared to patients with NTEMI, STEMI, and kidney failure at five-year follow-up.

**Figure 4:**
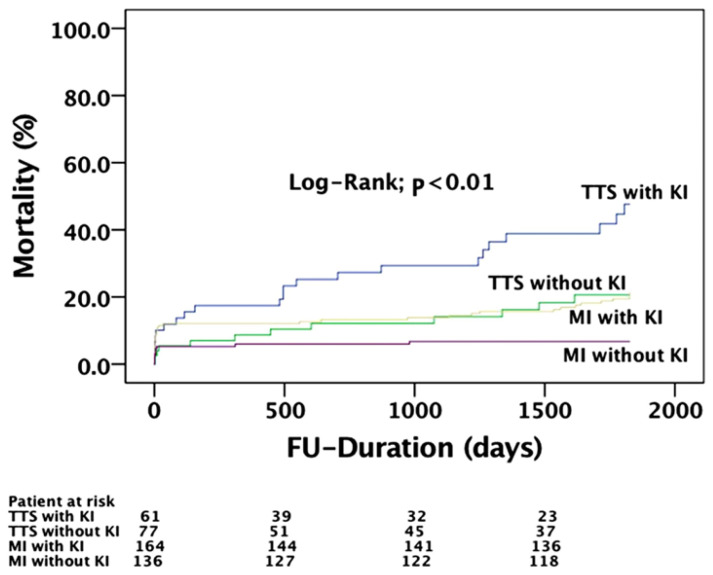
Mortality rate in patients suffering from takotsubo cardiomyopathy (TTS) with and without kidney failure compared to patients suffering MI with and without kidney failure at five-year follow-up.

There was a significantly higher rate of long-term mortality in the TTS group compared to MI (STEMI or NSTEMI), as seen in Figure 2 and Figure 3. In addition, we found a significantly higher mortality rate in patients with TTS and concomitant KF 4 years (29.5% vs. 15.9%, *p* = 0.02) and 5 years (34.4% vs. 20.7%, *p* = 0.03), Figure 4, after the index event compared to MI and concomitant KF. The difference in the long-term mortality rate was caused by non-cardiovascular reasons. In the TTS group, 7 patients died from cardiovascular events (4 cardiac arrest and 3 cardiogenic shock) and in the MI group 25 patients died from cardiovascular events (6 cardiac arrest, 15 cardiogenic shock, and 4 myocardial infarction). Non-cardiovascular reasons included pneumonia, sepsis, respiratory insufficiency, multiple organ failure, chronic obstructive pulmonary disease, lung cancer, breast cancer, anal carcinoma, leukemia, mesenteric ischemia, and kidney failure. There were no significant differences in thromboembolic events as well as in life-threatening arrhythmias and strokes between both groups at any time.

The rate of rehospitalization due to heart failure was higher in short-term, as well as long-term follow-up in patients with MI and KF (s. Table 3). The recurrence of MI after 4 and 5 years was higher than the recurrence of TTS (4.9% vs. 15.2% and 4.9% vs. 16.5%). In the cox univariate analysis, the male gender (HR 2.2, 95% CI 1.0–5.0, *p* = 0.04), EF < 35% (HR 2.1, 95% CI 1.1–4.3, *p* = 0.02), KF (HR 2.4, 95% CI 1.2–4.9, *p* = 0.01), cardiogenic shock (HR 4.6, 95% CI 2.2–9.3, *p* < 0.01), the use of inotropic drugs (HR 3.9, 95% CI 1.9–7.8, *p* < 0.01), and a history of cancer (HR 2.8, 95% CI 1.3–6.4, *p* < 0.01) were the predictors of five-year mortality (s. Table 4).

In a multivariate cox regression analysis male gender (HR 2.7, 95% CI 1.1–6.5, *p* = 0.02), KF (HR 2.8, 95% CI 1.2–6.0; *p* = 0.01) and history of cancer (HR 3.6, 95% CI 1.4–9.3; *p* < 0.01) were the independent predictors of five-year mortality (s. Table 4).

## 5. Discussion

In this retrospective study, a higher mortality rate was observed in patients with TTS and KF at long-term follow-up compared to patients with MI and KF. Also, the in-hospital need for invasive or non-invasive ventilation at the index event was more common in patients with TTS and KF.

Ando et al. previously demonstrated a worse outcome in patients suffering from TTS and KF [23,24]. The prevalence of KF and its determinants in coronary heart disease patients was analyzed in 24 European countries in the EUROASPIRE IV survey. They demonstrated a worse outcome for the association between coronary heart disease and KF. However, to our knowledge, there is no data comparing the influence of KF in MI and TTS. The in-hospital mortality of patients with TTS and KF may be related to a decline in left ventricular function, which was lower in these patients at the index event (EF 37% vs. 48%) compared to the MI patients. However, with the recovery of left ventricular function in follow-up, the in-hospital mortality was equalized. Interestingly the rate of rehospitalization because of heart failure, the rate of device implantation, and the prescription of ß-blockers and aspirin were higher and more common in patients with MI than in patients with TTS. It seems that the TTS patients may suffer more frequently from non-cardiovascular deaths. Of note, it has been reported that the cancer rate and other comorbidities are noted to be higher among patients with TTS [27,28].

Another observation was that the medical treatment differed in both groups. Aspirin and ß-blockers were prescribed more often in patients with MI and KF. The BEAT-AMI trial indicates a protective effect of intravenous ß-blocker therapy in patients with STEMI [29], probably because better heart rate control prevents a worsening of cardiac damage in ischemia. Nevertheless, the efficacy and safety of early intravenous beta-blockers in MI patients remains under question and randomized clinical trials are required [30,31]. For the acute phase of TTS, there is still no data and no standard therapy. Although, there is a lack of accurate understanding of the pathophysiologic mechanisms of TTS, catecholamines and microvascular dysfunction are understood to play an important role in the pathogenesis [8,32,33]. However, causality between the use of beta-blockers and/or aspirin among TTS with KF patients and the outcome has not been proven in prospective trials until today. There is a bevy of case reports that describe the development of an acute phase of TTS after the application of α- or β-agonists [29,34,35]. If a ß-agonist may provoke an acute phase of TTS, it seems to be consequent that a ß-blocker may impact the presence of the TTS pattern or/or outcome. Another case report indicates that antihypertensive therapy with intravenous administration of α-1-adrenergic blocker urapidil and ß-blocker bisoprolol at the highest recommended doses seemed to lead to a fast and full recovery of TTS [36]. Data from an animal model and human cardiomyocytes from induced pluripotent stem cells [37] support this theory. In summary, the reason for the higher mortality rate in patients with TTS and KF could be worse follow-up care and the relevant role of non-cardiovascular causes of death. Finally, it has been recommended that a combination of beta-blockers and angiotensin-converting enzyme inhibitors may have a more beneficial effect on TTS compared to each drug alone [38,39].

According to the current data, it seems that in patients with TTS and concomitant KF the risk of short- and long-term complications is higher compared to patients without. Therefore, physicians need to be aware of this issue and despite the reversibility of TTS, these patients must visit cardiologists frequently to avoid the underestimation of this high-risk group.

Our study had limitations; first, this was a single-center retrospective observational study including patients admitted over a period of 13 years.

Second, there is no information about the development of KF. Therefore, we have no information on the rate of acute or chronic KF. Of note, the eGFR was used as a cut in the present analysis for KF, whereas in the early stages of KF protein levels in urine need to be measured. However, the early stages of KF can only be diagnosed by measuring the protein levels in the urine. Third, the time frame for the inclusion of the MI patients is not completely the same as the TTS patients. This is related to the limited number of TTS (up to 5% among patients admitted to the Cath lab) patients and the subsequent need to include patients’ overall years to reach a feasible number of patients. The two study groups could be extremely asymmetric in number, which could affect any statistical conclusions drawn. All these aspects may affect the conclusions. Finally, regarding the retrospective character of this study, the bias is not excluded, e.g., in the case of evaluating the drugs used for a TTS event.

## 6. Conclusions

The higher mortality rate among TTS and KF underlines the importance of adequate and frequent follow-ups. The long-term mortality among TTS and KF could be predominated by non-cardiovascular causes of death.

## Figures and Tables

**Table 1:** Baseline characteristics of 61 patients initially presenting with takotsubo cardiomyopathy (TTS) and kidney failure (KF) and 164 patients presenting with myocardial infarction and KF.

Variables	TTS (*n* = 61)	MI (*n* = 164)	*p* Value *
Demographics			
Age. Mean ± SD	69 ± 11	71 ± 11	0.12
Female (%)	47 (77.0)	40 (24.4)	<0.01
Symptoms. *N* (%)			
Dyspnoe	24 (39.3)	53 (32.3)	0.32
Chest pain	31 (51.7)	118(72.0)	<0.01
Clinic parameter			
Systolic BP. mmHg	132 (90–200)	137 (80–250)	0.65
Diastolic BP. mmHg	77 (50–110)	77 (30–150)	0.55
Heart rate. Bpm	100 ± 25	79 ± 19	<0.01
ECG Data. *N* (%)			
ST-segment elevation	16 (26.2)	74 (45.1)	0.01
Inversed T-Waves	56 (93.3)	119 (72.6)	<0.01
PQ-interval	166 ± 28	173 ± 33	<0.01
QTc (ms)	468 (374–554)	448 (324–569)	0.53
Laboratory values. Mean ± SD			
Troponin I (U/L) (IQR)	48.13 (0.01–2631)	28.83 (0.01–1704)	0.50
Creatine phosphatkinase (U/L) (IQR)	993 (39–26,600)	909 (30–12,092)	0.39
CKMB (U/L) (IQR)	43 (2–415)	88 (0–1208)	0.34
C-Reactive protein (mg/L) (IQR)	61.3 (0.4–467.1)	34.3 (0.0–247.9)	0.34
Hemoglobin (g/dL) (IQR)	12.0 ± 2.4	13.2 ±2.4	0.03
Creatinine (mg/dL) (IQR)	1.52 (1.01–2.83)	1.50 (1.00–12.16)	0.38
Echocardiography data. *N* (%)			
LV EF%	37 ± 10	48 ± 14	<0.01
LV EF% follow-up	49 ± 14	48 ± 14	1.00
Mitral regurgitation	33 (54.1)	66 (40.2)	0.06
Tricuspid regurgitation	28 (45.9)	37 (22.6)	<0.01
Medical history. *N* (%)			
Smoking	15 (24.6)	60 (36.6)	0.09
Diabetes mellitus	13 (21.3)	65 (39.6)	0.01
BMI > 25 kg/m^2^	13 (26.0)	76 (46.3)	0.01
Hypertension	36 (59.0)	129 (78.7)	<0.01
COPD	11 (18.0)	7 (4.3)	<0.01
Atrial fibrillation	14 (23.0)	36 (22.0)	0.87
Coronary artery disease	12 (19.7)	137 (83.5)	<0.01
History of malignancy	8 (13.1)	11 (6.7)	0.12
Drugs on admission. *N* (%)			
Beta-blocker	18 (32.1)	79(48.5)	0.03
ACE inhibitor	19 (33.9)	63 (38.7)	0.53
Aldosteron inhibitor	1 (1.8)	2 (1.2)	1.00
ASS	17 (30.4)	74 (45.4)	0.05
Therapeutic anticoagulation	2 (3.6)	16 (9.8)	0.15
Drugs on discharge. *N* (%)			
Beta-blocker	42 (68.9)	131 (79.9)	0.08
ACE inhibitor	32(52.5)	104 (63.4)	0.14
Aldosteron inhibitor	0 (0.0)	5 (3.0)	0.33
Aspirin	26 (42.6)	139 (84.8)	<0.01
Therapeutic anticoagulation	16 (26.2)	15 (9.1)	<0.01

* *p* values for the comparison between TTS and MI; SD. Standard deviation; ECG. Electrocardiogram; EF. Ejection fraction; BMI. Body mass index. COPD. Chronic obstructive pulmonary disease; ACE. Angiotensin-converting enzyme.

**Table 2:** In-hospital events and treatment strategies in patients suffering from takotsubo cardiomyopathy (TTS) and KF compared to patients with myocardial infarction and kidney failure.

Variables	TTS (*n* = 61)	MI (*n* = 164)	*p* Value *
Life-threatening arrhythmia	9 (14.8)	29 (17.7)	0.60
NPPV and or intubation	40 (65.6)	24 (14.6)	<0.01
Inotropic agents	15(24.6)	26 (15.9)	0.13
Resuscitation	7(11.5)	25 (15.2)	0.47
ICD or pacemaker Implantation	4 (6.6)	44 (26.8)	<0.01
Admission to ICU. length of stay (IQR)	6 (0–52)	3 (0–31)	0.02
In-hospital death	6 (9.8)	19 (11.6)	0.71
Cardiogenic Shock	17 (27.9)	30 (18.3)	0.12

* *p* values for the comparison between TTS and MI; NPPV. Noninvasive positive pressure ventilation; ICU. Intermediate care unit.

**Table 3:** Outcomes in 61 TTS patients and 164 MI patients with kidney failure.

Variables	TTS (*n* = 61)	MI (*n* = 164)	Relative Risk (95% CI)	*p* Value *
In-hospital mortality	6 (9.8)	19 (11.6)	0.8 (0.4–2.0)	0.71
30-day mortality	5 (8.2)	19 (11.6)	0.7 (0.3–1.8)	0.46
Long-term mortality	23 (37.7)	34 (20.7)	1.8 (1.2–2.8)	0.02
Cardiovascular cause of death	7 (11.5)	26 (15.9)	0.7 (0.3–1.6)	0.41
Non-cardiovascular cause of death	12 (19.7)	5 (3.0)	6.5 (2.4–17.6)	<0.01
Unknown cause of death	4 (6.6)	4 (2.4)	8.8 (1.0–73.5)	0.22
30-day Stroke	1 (1.6)	1 (0.6)	2.7 (0.2–42.3)	0.47
1-year Stroke	2 (3.3)	1 (0.6)	5.4 (0.5–58.2)	0.18
Long-term Stroke	5 (8.2)	4 (2.4)	3.4 (0.9–12.1)	0.06
30-day life-threatening arrythmia	8 (13.1)	21 (12.8)	1.0 (0.5–2.2)	0.95
1-year life-threatening arrythmia	8 (13.1)	22 (13.4)	1.0 (0.5–2.0)	0.95
Long-term life-threatening arrythmia	8 (13.1)	36 (22.0)	0.6 (0.3–1.2)	0.14
30-day Heart Failure	3 (4.9)	27 (16.5)	0.3 (0.1–0.9)	0.02
1-year Heart Failure	4 (6.6)	33 (20.1)	0.3 (0.1–0.9)	0.02
Long-term Heart Failure	5 (8.2)	44 (26.8)	0.3 (0.1–0.7)	<0.01
30-day Recurrence	0 (0.0)	1 (0.6)		1.00
1-year Recurrence	0 (0.0)	10 (6.1)		0.07
Long-term Recurrence	3 (4.9)	27 (16.5)	0.3 (0.1–0.9)	0.03
30-day Thromboembolic Events	1 (1.6)	0 (0.0)		0.32
1-year Thromboembolic Events	1 (1.6)	0 (0.0)		0.27
Long-term Thromboembolic Events	2 (3.3)	5 (3.0)	1.0 (0.2–5.4)	1.00

* *p* values for the comparison between TTS and female MI patients.

**Table 4:** Univariate and multivariate analysis for primary endpoint.

Multivariate Analysis for the End Point						
	Univariate Analysis			Multivariate Analysis		
	HR	95%CI	*p*-Value	HR	95%CI	*p*-Value
Male	2.2	1.0–5.0	0.04	2.7	1.1–6.5	0.02
Age	1.0	0.9–1.0	0.45			
EF < 35%	2.1	1.1–4.3	0.02	1.3	0.5–2.9	0.49
COPD	1.1	0.4–2.4	0.85			
GFR < 60 mL/min	2.4	1.2–4.9	0.01	2.8	1.2–6.0	0.01
Cardiogenic shock	4.6	2.2–9.3	<0.01	2.7	0.6–11.8	0.18
Inotropic drugs	3.9	1.9–7.8	<0.01	1.25	0.2–6.1	0.77
DM Typ II	1.0	0.4–2.2	0.97			
Hypertension	0.7	0.3–1.5	0.41			
Apical ballooning	1.8	0.7–4.3	0.18			
History of cancer	2.8	1.3–6.4	<0.01	3.6	1.4–9.3	<0.01
Smoking	0.8	0.3–1.7	0.64			

HR, hazard ratio; EF, ejection fraction.

## Data Availability

All raw data will be available by request by the corresponding author.

## References

[1] Korlakunta H.L., Thambidorai S.K., Denney S.D., Khan I.A. (2005). Transient left ventricular apical ballooning: A novel heart syndrome. Int. J. Cardiol..

[2] Fazio G., Novo G., Azzarelli S., Evola S., Barbaro G., Sutera L., di Gesaro G., Akashi Y.J., Novo S. (2008). Transient mid-ventricular dyskinesia: A variant of Takotsubo syndrome. Int. J. Cardiol..

[3] El-Battrawy I., Santoro F., Stiermaier T., Moller C., Guastafierro F., Novo G., Novo S., Mariano E., Romeo F., Romeo F. (2021). Incidence and Clinical Impact of Right Ventricular Involvement (Biventricular Ballooning) in Takotsubo Syndrome: Results from the GEIST Registry. Chest.

[4] Templin C., Ghadri J.R., Diekmann J., Napp L.C., Bataiosu D.R., Jaguszewski M., Cammann V.L., Sarcon A., Geyer V., Neumann C.A. (2015). Clinical Features and Outcomes of Takotsubo (Stress) Cardiomyopathy. N. Engl. J. Med..

[5] Yasu T., Tone K., Kubo N., Saito M. (2006). Transient mid-ventricular ballooning cardiomyopathy: A new entity of Takotsubo cardiomyopathy. Int. J. Cardiol..

[6] Tsuchihashi K., Ueshima K., Uchida T., Oh-mura N., Kimura K., Owa M., Yoshiyama M., Miyazaki S., Haze K., Ogawa H. (2001). Transient left ventricular apical ballooning without coronary artery stenosis: A novel heart syndrome mimicking acute myocardial infarction. Angina Pectoris-Myocardial Infarction Investigations in Japan. J. Am. Coll. Cardiol..

[7] Dote K., Sato H., Tateishi H., Uchida T., Ishihara M. (1991). Myocardial stunning due to simultaneous multivessel coronary spasms: A review of 5 cases. J. Cardiol..

[8] Wittstein I.S., Thiemann D.R., Lima J.A., Baughman K.L., Schulman S.P., Gerstenblith G., Wu K.C., Rade J.J., Bivalacqua T.J., Champion H.C. (2005). Neurohumoral features of myocardial stunning due to sudden emotional stress. N. Engl. J. Med..

[9] Aweimer A., El-Battrawy I., Akin I., Borggrefe M., Mugge A., Patsalis P.C., Urban A., Kummer M., Vasileva S., Stachon A. (2021). Abnormal thyroid function is common in takotsubo syndrome and depends on two distinct mechanisms: Results of a multicentre observational study. J. Intern. Med..

[10] El-Battrawy I., Borggrefe M., Akin I. (2016). Endothelial dysfunction in takotsubo syndrome. Int. J. Cardiol..

[11] El-Battrawy I., Borggrefe M., Akin I. (2017). Hormone Status Correlates With Incidence of Heart Failure. J. Am. Coll. Cardiol..

[12] El-Battrawy I., Borggrefe M., Akin I. (2021). The current evidence of Takotsubo syndrome. Future Cardiol..

[13] Becher T., El-Battrawy I., Baumann S., Fastner C., Behnes M., Lossnitzer D., Elmas E., Hoffmann U., Papavassiliu T., Kuschyk J. (2016). Characteristics and long-term outcome of right ventricular involvement in Takotsubo cardiomyopathy. Int. J. Cardiol..

[14] El-Battrawy I., Lang S., Ansari U., Behnes M., Hillenbrand D., Schramm K., Fastner C., Zhou X., Bill V., Hoffmann U. (2017). Impact of concomitant atrial fibrillation on the prognosis of Takotsubo cardiomyopathy. Europace.

[15] El-Battrawy I., Lang S., Ansari U., Tulumen E., Schramm K., Fastner C., Zhou X., Hoffmann U., Borggrefe M., Akin I. (2017). Prevalence of malignant arrhythmia and sudden cardiac death in takotsubo syndrome and its management. Europace.

[16] Ionescu C.N., Aguilar-Lopez C.A., Sakr A.E., Ghantous A.E., Donohue T.J. (2010). Long-term outcome of Tako-tsubo cardiomyopathy. Heart Lung Circ..

[17] Stiermaier T., Thiele H., Eitel I. (2016). Prognosis in Patients With Takotsubo Cardiomyopathy. JACC Heart Fail..

[18] El-Battrawy I., Cammann V.L., Kato K., Szawan K.A., di Vece D., Rossi A., Wischnewsky M., Hermes-Laufer J., Gili S., Citro R. (2021). Impact of Atrial Fibrillation on Outcome in Takotsubo Syndrome: Data from the International Takotsubo Registry. J. Am. Heart Assoc..

[19] El-Battrawy I., Santoro F., Stiermaier T., Moller C., Guastafierro F., Novo G., Novo S., Santangelo A., Mariano E., Romeo F. (2020). Prevalence, management, and outcome of adverse rhythm disorders in takotsubo syndrome: Insights from the international multicenter GEIST registry. Heart Fail. Rev..

[20] El-Battrawy I., Behnes M., Hillenbrand D., Haghi D., Hoffmann U., Papavassiliu T., Lang S., Fastner C., Becher T., Baumann S. (2016). Prevalence, Clinical Characteristics, and Predictors of Patients with Thromboembolic Events in Takotsubo Cardiomyopathy. Clin. Med. Insights Cardiol..

[21] Gili S., Cammann V.L., Schlossbauer S.A., Kato K., D’Ascenzo F., di Vece D., Jurisic S., Micek J., Obeid S., Bacchi B. (2019). Cardiac Arrest in Takotsubo Syndrome: Results from the InterTAK Registry. Eur Heart J..

[22] El-Battrawy I., Gietzen T., Ansari U., Behnes M., Lang S., Zhou X., Borggrefe M., Akin I. (2018). Short-term and long-term incidence of stroke in Takotsubo syndrome. ESC Heart Fail..

[23] Ando K., Sukekawa H., Takahata A., Kobari Y., Tsuchiya H., Ishigaki D., Tamabuchi T., Koyama Y. (2017). Renal dysfunction indicative of outcomes in hospitalized patients with takotsubo syndrome. Eur Heart J. Acute Cardiovasc. Care.

[24] Bill V., El-Battrawy I., Hoffmann U., Haghi D., Kuschyk J., Borggrefe M., Akin I. (2018). Takotsubo Cardiomyopathy: Another Form of Cardiorenal Syndrome. Angiology.

[25] Marenzi G., Cabiati A., Bertoli S.V., Assanelli E., Marana I., de Metrio M., Rubino M., Moltrasio M., Grazi M., Campodonico J. (2013). Incidence and relevance of acute kidney injury in patients hospitalized with acute coronary syndromes. Am. J. Cardiol..

[26] Madhavan M., Prasad A. (2010). Proposed Mayo Clinic criteria for the diagnosis of Tako-Tsubo cardiomyopathy and long-term prognosis. Herz.

[27] Cammann V.L., Sarcon A., Ding K.J., Seifert B., Kato K., di Vece D., Szawan K.A., Gili S., Jurisic S., Bacchi B. (2019). Clinical Features and Outcomes of Patients With Malignancy and Takotsubo Syndrome: Observations from the International Takotsubo Registry. J. Am. Heart Assoc..

[28] Pelliccia F., Parodi G., Greco C., Antoniucci D., Brenner R., Bossone E., Cacciotti L., Capucci A., Citro R., Delmas C. (2015). Comorbidities frequency in Takotsubo syndrome: An international collaborative systematic review including 1109 patients. Am. J. Med..

[29] Lee A., Nguyen P. (2016). Takotsubo Cardiomyopathy Due to Systemic Absorption of Intraocular Phenylephrine. Heart Lung Circ..

[30] Collet J.P., Thiele H., Barbato E., Barthelemy O., Bauersachs J., Bhatt D.L., Dendale P., Dorobantu M., Edvardsen T., Folliguet T. (2021). 2020 ESC Guidelines for the management of acute coronary syndromes in patients presenting without persistent ST-segment elevation. Eur. Heart J..

[31] Ibanez B., James S., Agewall S., Antunes M.J., Bucciarelli-Ducci C., Bueno H., Caforio A.L.P., Crea F., Goudevenos J.A., Halvorsen S. (2018). 2017 ESC Guidelines for the management of acute myocardial infarction in patients presenting with ST-segment elevation: The Task Force for the management of acute myocardial infarction in patients presenting with ST-segment elevation of the European Society of Cardiology (ESC). Eur. Heart J..

[32] Pelliccia F., Kaski J.C., Crea F., Camici P.G. (2017). Pathophysiology of Takotsubo Syndrome. Circulation.

[33] Lyon A.R., Bossone E., Schneider B., Sechtem U., Citro R., Underwood S.R., Sheppard M.N., Figtree G.A., Parodi G., Akashi Y.J. (2016). Current state of knowledge on Takotsubo syndrome: A Position Statement from the Taskforce on Takotsubo Syndrome of the Heart Failure Association of the European Society of Cardiology. Eur. J. Heart Fail..

[34] Khwaja Y.H., Tai J.M. (2016). Takotsubo cardiomyopathy with use of salbutamol nebulisation and aminophylline infusion in a patient with acute asthma exacerbation. BMJ Case Rep..

[35] Rasiah S., Finger R.P., MacIsaac A.I., Lim L.L. (2016). Takotsubo syndrome caused by subconjunctival injection of a mydricaine analogue. Clin. Exp. Ophthalmol..

[36] Eitel I., Lucke C., Behrendt F., Sareban M., Gutberlet M., Schuler G., Thiele H. (2010). Full recovery of Takotsubo cardiomyopathy (apical ballooning) in two days. Int. J. Cardiol..

[37] Huang M., Fan X., Yang Z., Cyganek L., Li X., Yuecel G., Lan H., Li Y., Wendel A., Lang S. (2021). Alpha 1-adrenoceptor signalling contributes to toxic effects of catecholamine on electrical properties in cardiomyocytes. Europace.

[38] Ishikura F., Takano Y., Ueyama T. (2012). Acute effects of beta-blocker with intrinsic sympathomimetic activity on stress-induced cardiac dysfunction in rats. J. Cardiol..

[39] Brunetti N.D., Santoro F., de Gennaro L., Correale M., Gaglione A., di Biase M., Madias J.E. (2017). Combined therapy with beta-blockers and ACE-inhibitors/angiotensin receptor blockers and recurrence of Takotsubo (stress) cardiomyopathy: A meta-regression study. Int. J. Cardiol..

